# Supplementary material for: Individual and system causes of moral distress experienced by public health practitioners in Canada
Source: PLOS Glob Public Health. 2026 Nov 4;6(11):e0006673. doi: 10.1371/journal.pgph.0006673 (PMC13734438; doi:10.1371/journal.pgph.0006673)
Supplement: Supplementary file 3 [file pgph.0006673.s003.docx]

# **S1 Table – Discussion Guide**

| **Key areas of investigation** | **Topic** |
| --- | --- |
| Rapport building  warm up | **Introduction** |
|  | - Thank them for their time. - Inquiring about how they learned about the study. - Introducing the researcher, their background. - Reviewing the consent documents, their rights during the interview and available support if needed. - Confirming confidentiality of interview and their responses. - Inquiring if they had any questions about the study or consent process. - Confirm they agree to recording of the interview. |
| Participant Details | **Socio-demographic details** |
|  | **Age Bracket**; 18-24; 25-34; 35-44; 45-54; 55-64; 65 or over **Gender** \|; Female \| Trans female; Male \| Trans male; Non-Binary \| Not listed; Do not know \| Prefer not to say The following question is from the Canadian Institute for Health Information: In our society, people are often described by their race or racial background. These are not based in science, but our race may influence the way we are treated by individuals and institutions, and this may affect our health. Which category(ies) best describes you? Include all that apply. Response category \| Examples; Black \| African, African Canadian, Afro-Caribbean descent; East Asian \| Chinese, Japanese, Korean, Taiwanese descent; Indigenous (First Nations, Inuk/Inuit, Métis) \| First Nations, Inuk/Inuit, Métis descent; Latin American \| Hispanic or Latin American descent; Middle Eastern \| Arab, Persian, West Asian descent (e.g. Afghan, Egyptian, Iranian, Kurdish, Lebanese, Turkish); South Asian \| South Asian descent (e.g., Bangladeshi, Indian, Indo-Caribbean, Pakistani, Sri Lankan); Southeast Asian \| Cambodian, Filipino, Indonesian, Thai, Vietnamese, or other Southeast Asian descent; White \| European descent; Another race category Optional: \| Includes values not described above; Do not know \| Not applicable; Prefer not to answer \| Not applicable |
|  | **Experience and Education** |
|  | - Years of experience as a healthcare practitioner? - Years of experience in Public/Community/Health Promotion Practice? - Educational background? |
| Area of practice in public health.  Understanding of public health work. | **Area and location of work experience** |
|  | - Different practice locations and focus could impact work and experiences.   Prompts:   - Where have you worked in public health (local/municipal level, provincial -Rural vs urban? National work, international work. Not asking specific organization). - Level or reported health promotion activity, micro-macro. - Establish if participant is primarily in health promotion, disease/injury prevention or other public health arenas. - Explore if participant views their public health work as individually based or systemic work.   Prompts   - What work, or what teams have you worked for in public health? - Can you explain to me a bit about your day-to-day work and how you do it? (work in-person with patients, work over the phone with patients? Work in an office, clinic, community spaces?) - What have been your primary roles in work? (Identify work on health promotion continuum. ex: work primarily with patients directly, upstream work with institutional policies/procedures, systemic work with politics/ building healthy public policy, other) |
| Understanding of moral distress | **Definition of Moral Distress** |
|  | - Inquiry into understanding of moral distress.   Prompt:   - What do you understand moral distress to be?   Provided the definition: “When one knows the right thing to do, but institutional constraints make it nearly impossible to pursue the right course of action” (1) Jameton, 1984  Provided further definitions if needed.   - When the practitioner feels certain of the ethical course of action but is constrained from taking that action (26) Hamric and Blackhall |
| Experiences of moral distress | **Experiences of moral distress, including ethical dilemmas contributing to moral distress** |
|  | Prompt   - Can you think of any public health work experience you have that you believe caused you moral distress? - Can you explain to me what the situation was? |
| Moral Compassing of moral distress experiences | **Emotions, Sources, Constraints, Responsibilities and Actions** |
|  | Prompts:   - Were there any emotions you felt? What emotions did you feel? - What was the source of the moral distress? (ex: did you not act due to feelings vs did you try to act but felt constrained) - What internal or external factors did you experience/feel in the situation? (Constraints such as patient didn’t qualify for services needed, internal fears that your concerns were ignored) - Who was responsible for what in this situation? - Where there any conflicting responsibilities/values/norms/interests (ethical dilemmas)? (ex: you (X) have certain values/responsibilities/ obligations but those conflicted with (Y… the patient etc) values/ obligations/responsibilities). - What, if any, actions could you take? What actions did you take? - What action do you feel you should have, or wished you had taken? - Is there anything else you would like to discuss? |
| Concluding remarks | **Ending the Interview** |
|  | - Inquiry, if there were any further areas they wished to discuss. - Thanked them for their time. - Inquired if any supports for mental health were needed. Provided supports as needed. |
