# Supplementary material for: Individual and system causes of moral distress experienced by public health practitioners in Canada
Source: PLOS Glob Public Health. 2026 Nov 4;6(11):e0006673. doi: 10.1371/journal.pgph.0006673 (PMC13734438; doi:10.1371/journal.pgph.0006673)
Supplement: Supplementary file 4 [file pgph.0006673.s004.docx]

**S2 Table – Codes, Subthemes and Themes of data**

| **Code** | **Subthemes (1^st^ order)** | **Subthemes (2^nd^ order)** | **Main themes** |
| --- | --- | --- | --- |
| Regulatory Body | Regulatory body direction may contradict work direction. | Moral distress related to powerlessness in public health structures | Systemic Powerlessness |
| Supportive management | Management can have a positive impact on work. |  |  |
| Lacking management | When management is non-supportive, it can make a situation of moral distress more significant. |  |  |
| Responsibility | Feelings of professional requirements we need to fulfill. |  |  |
| Competence | May feel distress if there is a colleague perceived as being incompetent, or if the care that is given is at a lower threshold due to staff knowledge/ experience. |  |  |
| Better understanding/ communication | If better communication was done, it could lead to better understanding. Amplified during the pandemic. |  |  |
| Resource limitation | Resource constraints on work that can be done. |  |  |
| Bottom up approach | When grass roots project or movements are discussed or going from bottom to top with work and projects. |  |  |
| Top-down approach | Hierarchal structures or power to influence and change. Being in positions of authority can give more power to affect change. Being in a position with less authority or ability to affect change can lead to feelings of moral distress or not being heard. |  |  |
| Restructuring | Large organizational changes that make goals and reporting confusing. |  |  |
| Removing autonomy | Taking away people’s choice for collective good - it can cause moral distress to implement these mandates without a supportive culture or community. |  |  |
| Population level | Health impacts at the population level. |  |  |
| Narrow view | Limitations on the work that can be done not due to resources, but more based on differing views within an organization | Limited scope of care you can provide, not due to reasons of resources or competence. |  |
| Scope limits | Being limited from providing services and care for reasons not related to competence or resources. |  |  |
| Politics | Politics impacting the healthcare that can be provided. |  | Political and ideological overreach |
| Lacking logic | Decisions are made that have no clear reasons, including political reasons. |  |  |
| Job threats | Employers use job loss/firing/not renewing contracts to minimize advocacy | Suppressed  as a way to control staff, minimize advocacy, employers threaten job loss, firing, and not renewing contracts, as well as reprimands short of job loss.  Having your voice stifled; being forced to act or not act either due to colleagues or external forces. |  |
| Reprimands | Getting into trouble with employer based on decisions and actions taken |  |  |
| Silenced | Being unable to share information or not do certain work. |  |  |
| Intimidation | Either internal or external pressures to try to get you to act or not act in a certain way. |  |  |
| Standing ground | Not backing down with advocacy, despite reprimands or threats. |  |  |
| Have and have nots | Some areas are better funded or resourced than others across teams and organizations. |  |  |
| Public health doing non public health work | Public health has many roles, but at times it may be asked to do work that should sit with other healthcare areas – which uses its resources to do nonpublic health work. |  |  |
| System failures | When healthcare providers are working with patients or within the system, but encounter barriers in the system, such as nowhere to refer someone with no health card, or budget cuts that create weaknesses, these are system failures. |  |  |
| Misinformation | Publishing inaccurate or conflicting information to the population – amplified during the pandemic. | Constraints during the pandemic |  |
| Covid changes | The way of working has changed with covid, and some areas are returning to normal, other areas are remaining changed. |  |  |
| Health promotion | Work that looks to prevent or promote health. | Public Health Span  Public health is prevention-focused work that spans a continuum from individual care to population health. | Unethical work environments and undervalued expertise |
| Public Health Scope | What public health work should be, the scope of public health work. |  |  |
| Circumnavigating | Trying to affect change, not always following the rules – going around the systems in a way to try to do what is felt to be right. | Unethical work environment |  |
| Advocacy | Trying to affect change by following the rules. |  |  |
| Mitigation | Aim to reduce negative impacts when we can’t prevent them. |  |  |
| Optics | Organizations talk only about doing something instead of implementing it. |  |  |
| Covid priority | Having all resources pulled to the covid response, at the expense of vulnerable communities with equally valid health concerns. |  |  |
| Irresponsible spending | Witnessing seemingly bad decisions around spending and budget. |  |  |
| Not respecting expertise | When education or knowledge are not accepted or not used. |  |  |
| Workplace injury | Injury or illness from work/workplace. |  |  |
| Team dynamics | Hierarchy, inter team and interprofessional interactions can impact work and outcomes. Team dynamics can create distress and moral distress, especially if there are differing values and/or degrees of authority. Team dynamics can also be supportive. | Workplace dynamics  How people work with or respond within a team and turn to a colleague in difficult times. |  |
| Workplace support | Turning to colleagues in difficult times |  |  |
| Health equity | Focusing work to help those most in need. |  |  |
| Inequity | Individuals experience differences in their work environment based on factors including race. Also, population level interventions disadvantage some communities in systematic ways. | Unethical work  Work in public health that disadvantages some communities. Experiencing inequity within the work environment based on different factors including race. |  |
| Racism | When someone is treated differently because of race |  |  |
| Distress – not moral distress | Distress when not involving values or morals. |  |  |
| Work experience | The more experience one has, the more confidence they have in their own work, and more comfortable they are with difficult experiences and decisions. |  |  |
| Small differences | Making impacts at the individual level. |  |  |
| Public health pride | Many practitioners in public health explicitly chose to work in public health and feel pride in their work in this field. |  | Moral injury |
| Value lost | Many people have experience and knowledge in public health, however it isn't always recognized or used, which can motivate staff to leave. Not being able to contribute to work they would be well experienced to contribute to, and want to contribute to. |  |  |
| Cultural differences | When the organization has different cultures and values than you. |  |  |
| Witness | Being a witness to system failures, errors or things that have negative impacts on patients. | Violations or witnessing violations to things you know are right |  |
| True consent | Healthcare practitioners must get consent from their clients, but sometimes it doesn't feel like true consent is given because of systemic issues, or other broad challenges that impact the consent process. |  |  |
| Settling | Accepting things that you don’t want to have to accept. |  |  |
| Coping | Strategies to manage difficult events/situations. |  |  |
| Patient impacts | When there aren't enough resources, competence, leadership skills then this can impact the people and populations we are trying to care for. | Impacts on patients and populations |  |
| Mistrust in public health | Population doesn’t trust public health. |  |  |
| Moral injury | Moral injury has defined symptoms and impacts. It can be caused by cumulative or one-time incidences. It is different from burnout. |  |  |
| Emotions | Moral distress leads to different emotions, or different emotions lead to moral distress. |  |  |
| Public health misunderstood | The work that public health does is often not seen and not well understood. |  |  |
| Giving everything | Working all the time, giving up personal lives for work. | Giving away everything, forced to do things. |  |
| Can’t escape | When work spills into regular life and you feel like you can't escape it. |  |  |
| Can’t say no | Being forced to do work you didn't agree to do, or not being able to say no out of fear such as no one else can do the job, there are no staff to offer reprieve. |  |  |
| Sense of failure | When one doesn’t feel good about the work they are doing. |  |  |
| Leaving profession/job | Leaving work and jobs that they are trained and possibly licensed to do. |  |  |
| Can’t save the world | When one accepts that you can only do so much for a person, community, or population. |  |  |
